# Projection of Cortical Beta Band Oscillations to a Motor Neuron Pool across the Full Range of Recruitment

**DOI:** 10.1523/JNEUROSCI.0453-25.2025

**Published:** 2025-07-09

**Authors:** Emanuele Abbagnano, Alejandro Pascual-Valdunciel, Blanka Zicher, Jaime Ibañez, Dario Farina

**Affiliations:** ^1^Department of Bioengineering, Imperial College London, London W12 0BZ, United Kingdom; ^2^BSICoS Group, I3A Institute and IIS Aragón, University of Zaragoza, Zaragoza 50018, Spain; ^3^Centro de Investigación Biomídica en Red en Bioingeniería, Biomateriales y Nanomedicina (CIBER-BBN), Zaragoza 50018, Spain

**Keywords:** beta oscillations, corticomuscular coherence, corticospinal transmission, cumulant density analysis, motor neurons, motor unit recruitment

## Abstract

Cortical beta band oscillations (13–30 Hz) are associated with sensorimotor control, but their precise role remains unclear. Evidence suggests that for low-threshold motor neurons (MNs), these oscillations are conveyed to muscles via the fastest corticospinal fibers. However, their transmission to MNs of different sizes may vary due to differences in the relative strength of corticospinal and reticulospinal projections across the MN pool. Consequently, it remains uncertain whether corticospinal beta transmission follows similar pathways and maintains consistent strength across the entire MN pool. To investigate this, we examined beta activity in MNs innervating the tibialis anterior muscle across the full range of recruitment thresholds in a study involving 12 participants of both sexes. We characterized beta activity at both the cortical and motor unit (MU) levels, while participants performed contractions from mild to submaximal levels. Corticomuscular coherence remained unchanged across contraction forces after normalizing for the net MU spike rate, suggesting that beta oscillations are transmitted with similar strength to MNs, regardless of size. To further explore beta transmission, we estimated corticospinal delays using the cumulant density function, identifying peak correlations between cortical and muscular activity. Once compensated for variable peripheral axonal propagation delay across MNs, the corticospinal delay remained stable, and its value (∼14 ms) indicated projections through the fastest corticospinal fibers for all MNs. These findings demonstrate that corticospinal beta band transmission is determined by the fastest pathway connecting in the corticospinal tract, projecting similarly across the entire MN pool.

## Significance Statement

Beta band oscillations (13–30 Hz) play a key role in sensorimotor control, yet their precise transmission to motor neurons (MNs) remains unclear. This study demonstrates that beta oscillations are transmitted similarly across the entire MN pool, regardless of recruitment threshold. By examining corticomuscular coherence and corticospinal delays during voluntary contractions, we show that beta activity is consistently relayed to MNs via the fastest corticospinal fibers. These findings provide evidence that beta band activity is not preferentially directed toward specific subsets of MNs but is instead a global signal influencing motor output. This insight advances our understanding of how the central nervous system regulates movement and may have implications for neurorehabilitation and brain–machine interfaces.

## Introduction

Beta oscillations (13–30 Hz) are a prominent neural rhythm in the sensorimotor cortex and are associated with steady motor states and sensorimotor integration (Baker, 2007; Kilavik et al., 2013). These oscillations are transmitted to muscles during sustained motor tasks, as shown by corticomuscular coherence (CMC) analysis (Conway et al., 1995; Baker, 2007; Witham et al., 2011). This coupling between the cortex and muscles in the beta band indicates phase synchronization between cortical and muscular activity, evident especially during isometric contractions (Baker et al., 1997; Salenius et al., 1997 ; Echeverria-Altuna et al., 2022). Recent studies have shown that beta oscillations are transient rather than sustained neural signals, appearing as phase-coupled brief bursts at both cortical and muscular levels (Little et al., 2019; Bonaiuto et al., 2021; Echeverria-Altuna et al., 2022).

While it has been proposed that peripheral beta activity does not directly influence volitional force modulation, further investigation into its broader role in motor control is needed (Zicher et al., 2023, 2024). Previous work has shown that these oscillations exhibit abnormal corticomuscular coupling in neurological injuries associated with motor impairments (Engel and Fries, 2010), such as Parkinson's disease, stroke, and spinal cord injuries, reflecting disrupted communication between the cortex and muscles (Gourab and Schmit, 2010; Von Carlowitz-Ghori et al., 2014; Zokaei et al., 2021). Thus, while the exact function of peripherally transmitted beta oscillations remains uncertain, their transmission is likely crucial for motor control (Baker et al., 1999).

Cortical beta oscillations are transmitted through the corticospinal tract, with signals propagating along descending axons and summing at the soma of lower motor neurons (MNs; Baker et al., 2003). The conduction delays in this tract suggest that, at least for low-threshold MNs, beta transmission is determined by the fastest pathways in the corticospinal tract, as these contribute most significantly to the synchronization observed between cortical and peripheral beta rhythms (Ibáñez et al., 2021). However, while it is known that the input to a MN pool is largely shared across all MNs at “low” frequencies (Farina et al., 2014), which are associated with force control, it remains unclear whether beta band inputs follow the same distribution and pathways across different MN sizes.

Lower MNs within a pool vary in size and electrophysiological properties, following the size principle during voluntary activation (Henneman, 1957; Heckman and Enoka, 2004). While beta oscillations have been shown to be transmitted to peripheral muscles via low-threshold MNs, which are primarily involved in fine motor control, their transmission to high-threshold MNs engaged in strong contractions remains unclear. These units appear to be more influenced by the reticulospinal than the corticospinal tract (Baker, 2011; Glover and Baker, 2020). Accordingly, previous studies have reported a decrease in CMC values within the beta band as contraction force increases (Ushiyama et al., 2012; Cremoux et al., 2017; Dal Maso et al., 2017). This decline may indicate either a reduction in the common beta band input at higher contraction forces or a different projection of beta on small and large MNs. However, standard electromyography (EMG) and CMC analyses face limitations due to amplitude cancellation at high forces (Day and Hulliger, 2001; Keenan et al., 2006; Farina et al., 2008). These limits can be partially overcome by decomposing the EMG signal into the discharge activations of individual motor units (MUs; Farina and Holobar, 2016).

In this study, we characterized the projection of beta band oscillations to the pool of spinal MNs innervating the tibialis anterior (TA) muscle. We first demonstrated that we could identify the activity of MNs across a large range of recruitment thresholds. Despite the differences in thresholds, our results showed that beta oscillations were transmitted to all MNs using the fastest corticospinal fibers and with an approximately unvarying strength, irrespective of the MN size. These results provide novel insights into the transmission of beta oscillations to spinal MNs, which could ultimately contribute to elucidating the role of beta oscillations in motor control.

## Materials and Methods

### Experimental data acquisition

#### Subjects

Twelve healthy participants (ages, 28 ± 4.63 years; 10 males and 2 females) with no known history of neurological or musculoskeletal disorders were recruited for this study. While this sample size is comparable to previous studies (Ushiyama et al., 2012; Bräcklein et al., 2022; Zicher et al., 2024), the analysis method we employed provided a more precise assessment of corticospinal transmission by directly analyzing individual MUs rather than relying solely on global interference EMG (see below description of methods). This granular analysis reduces variability and improves sensitivity in detecting differences, leading to highly consistent and reliable findings, as demonstrated in the Results section. All participants provided written informed consent prior to their inclusion. The study was conducted in accordance with the principles outlined in the Declaration of Helsinki and received approval from the Imperial College London Ethics Committee (reference number 18IC4685).

#### Experimental paradigm

**Figure 1.**
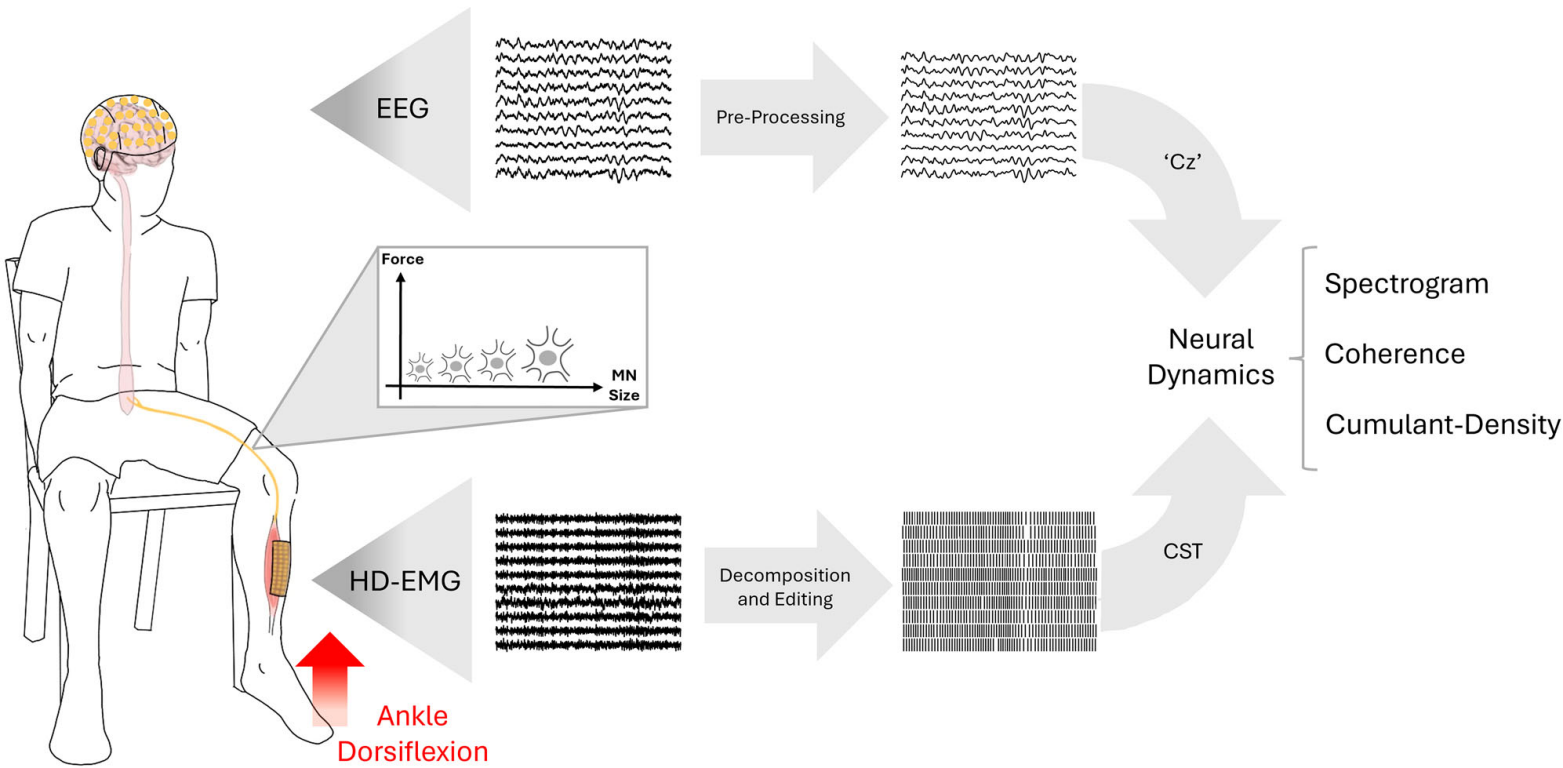
Overview of the experimental paradigm. Subjects performed multiple isometric dorsiflexion contractions using the TA muscle to investigate the transmission of cortical beta band oscillations to the muscle. Contractions were performed at 5, 10, 20, 30, 50, and 70% of the MVC, progressively recruiting larger MNs in accordance with Henneman's size principle. During each contraction, EEG and HD-EMG were recorded simultaneously to examine transmission across different MN sizes. EEG signals were filtered off-line, artifacts were removed, and the “Cz” channel was selected for further analysis. HD-EMG was decomposed into individual MU spike trains, from which the CST was computed. Transmission of cortical beta band oscillations between the cortex and muscle was then analyzed using spectrograms, coherence, and cumulant density.

To investigate the transmission of beta band activity across the MN pool, it is essential to study a broad range of MNs, which vary in size and recruitment thresholds. Larger MNs have higher thresholds than smaller MNs, thus requiring larger excitatory input to initiate firing (Henneman, 1957). Therefore, the experimental design involved subjects performing isometric contractions at progressively increasing forces, determining the recruitment of MNs across a large range of forces (Henneman, 1957). Isometric contractions were selected because they enhance beta band power both cortically and peripherally (Baker, 2007; Engel and Fries, 2010) while also facilitating the identification of individual MU firings at a steady rate. Cortical activity was measured using electroencephalography (EEG), while high-density surface EMG (HD-sEMG) was used to record signals from the TA muscle. The experimental paradigm is visualized in Figure 1.

#### Experimental design

Participants were seated comfortably in a chair with their knee flexed at ∼75° and their leg securely fixed to an ankle dynamometer using straps. The foot was positioned on a pedal inclined at 30° in the plantarflexion direction (where 0° corresponds to the foot being perpendicular to the shank).

At the start of the session, participants performed a single maximum voluntary contraction (MVC) of the ankle with a dorsiflexion to determine their individual MVC, which served as a reference for the subsequent tasks. Visual feedback was provided throughout the experiment, with a target displayed on a screen representing the required force level and a real-time trace showing the force produced by the participant.

Each participant performed a total of 16 isometric dorsiflexion contractions with the TA. These included a linear ramp phase where force increased from 0% to the target MVC level at a rate of 5% MVC per second, followed by a plateau phase at the target level, and finally a ramp-down phase back to 0% MVC at the same rate (trapezoidal force profile). Six target MVC levels were used: 5, 10, 20, 30, 50, and 70%. For the lower targets (5, 10, 20, and 30%), the plateau phase lasted 60 s. To minimize fatigue, contractions at 50 and 70% MVC were subdivided into multiple repetitions: five repetitions of 15 s for 50% MVC and seven repetitions of 10 s for 70% MVC. The duration of these plateaus was chosen based on previous studies showing successful decomposition for segments longer than 5 s at submaximal contraction levels (Del Vecchio et al., 2019; Avrillon et al., 2024b). The sequence of these contractions was randomized across participants. There was a 2 min rest period in between each contraction to reduce fatigue.

#### Data acquisition

HD-sEMG signals were recorded from the TA muscle of the self-reported dominant leg using a 256-electrode grid (10 columns × 26 rows, gold-coated, 1 mm electrode diameter, 4 mm interelectrode spacing; OT Bioelettronica). The electrode array was positioned over the muscle belly, aligned with the muscle fibers direction. EMG signals were recorded in monopolar derivation, amplified using the Quattrocento Amplifier system (OT Bioelettronica), sampled at 2,048 Hz and digitally bandpass filtered between 10 and 500 Hz. Force data were collected using a transducer (TF-022, CCt Transducer s.a.s.) mounted on the pedal of the ankle dynamometer and digitalized at 2,048 Hz using the Quattrocento Amplifier system.

EEG signals were recorded concurrently using 31 active gel-based electrodes placed according to the international 10–20 system (actiCAP, Brain Products). The FCz channel served as the online reference. EEG signals were amplified with the BrainVision actiCHamp Plus system (Brain Products), sampled at 1,000 Hz, and subsequently resampled to 2,048 Hz.

To ensure precise temporal alignment, all recordings were synchronized using a shared digital trigger signal sent to both the Quattrocento Amplifier system and the BrainVision actiCHamp Plus.

### Data analysis

#### HD-sEMG decomposition and processing

HD-sEMG signals recorded during the experimental tasks were off-line decomposed to identify the activity of individual MUs using a convolutive blind source separation algorithm (Negro et al., 2016). To ensure high-quality data, spike trains with a silhouette value (SIL) below 0.9 were automatically discarded (Holobar et al., 2014; Negro et al., 2016). The remaining spike trains were then manually reviewed to correct errors, following established guidelines (Del Vecchio et al., 2020). Specifically, missed firings that the peak detection algorithm failed to identify (Holobar and Zazula, 2007a,b; Negro et al., 2016) were added, and false positives—such as spikes that unrealistically increased the MU's firing rate during isometric contractions—were removed. This manual editing step, which depends on the operator's expertise, was used to improve MU identification in the next processing phase. Finally, we refined the spike train estimates by recalculating the MU separation filters and applying them again to the raw EMG data.

MU properties were extracted using a validated MATLAB script (Avrillon et al., 2024b). Key properties included the average smoothed firing rate and the recruitment threshold, defined as the percentage of MVC at which the MU began firing. The average firing rate was computed by taking the mean of the instantaneous firing rates, each calculated from the time intervals between consecutive spikes. The recruitment threshold was estimated by averaging 25 samples around the first firing time of the MU during the ramp-up phase (i.e., when the MU was first recruited), to minimize the impact of noise in the force signal.

To obtain longer spike trains for analysis at 50 and 70% MVC, individual MUs were tracked across repetitions. Tracking was performed using a validated MATLAB algorithm that leveraged the unique spatial distribution of MU action potential (MUAP) waveforms (Avrillon et al., 2024b). Pairing between MUs in different contractions was retained only if their MUAP cross-correlation exceeded 0.9 (Martinez-Valdes et al., 2017). However, regardless of the cross-correlation value, pairs were rejected if recruitment thresholds differed by >10% MVC, as this indicated a potential mismatch. After tracking, spike trains from successfully matched MUs across repetitions were merged.

Following these procedures, additional quality control criteria were applied. MUs were excluded if their SIL was below 0.9, their firing duration was <50% of the task time, or their instantaneous firing rate had a standard deviation (SD) exceeding 20 Hz (Negro et al., 2016; Del Vecchio et al., 2020). Decomposition and manual editing were conducted using the MUedit MATLAB application (Avrillon et al., 2024a). Only MUs meeting all quality criteria were retained for further analysis.

Because of the variability in the decomposition algorithm to define the discharge time, discharges were realigned to the MUAP onset, using the methods described in Ibáñez et al. (2021).

Following decomposition, the individual MU spike train was represented as a binary signal, where a value of 1 indicated a firing event. By summing these binary signals across a pool of decomposed MUs, a cumulative spike train (CST) was obtained, providing an estimate of the neural drive conveyed by MNs to the muscle (Farina and Negro, 2015). For each contraction level, a distinct CST was computed, resulting in six CSTs per participant, one for each contraction level.

To ensure that CSTs from different contraction levels, which may differ in the number and properties of contributing MUs, were comparable when used in the following coherence analyses (see below), it was necessary to standardize the amplification of the common input projected to the MN pool. In fact, the amplification gain of the common input in a CST is determined by the total number of MU firings, which is given by the number of recruited MUs and their firing rates (Farina et al., 2014). To address this issue, we normalized CSTs by ensuring that the total number of firings was matched across all contraction levels for each participant. Specifically, the contraction level with the lowest total number of firings was identified, and MUs were randomly selected from other levels to match this firing count. This random selection was repeated 10 times for each contraction level, generating 10 permutations of CSTs. In most cases, the contraction levels with the lowest firing counts—where no permutations could be performed—were 50 or 70% MVC. However, results indicated that variance remained consistent across contraction levels, suggesting that this normalization did not introduce significant differences in variability of the estimates.

All subsequent analyses were conducted on these CSTs, and the final values reported for each subject and contraction level were computed as the average across the 10 permutations.

#### EEG processing

The preprocessing of EEG signals was performed using the FieldTrip toolbox (Oostenveld et al., 2011). Signals were first off-line rereferenced to the average of the earlobe electrodes recorded during the experiment. A fourth-order Butterworth bandpass filter was applied to retain frequencies between 0.5 and 45 Hz. Independent component analysis was then utilized to identify and remove artifacts caused by eyeblinks, oculomotor activity, muscle activity, and other noise sources. To further enhance signal quality, a surface Laplacian filter was applied to eliminate common inputs across electrodes using the CSD Toolbox (Kayser and Tenke, 2006).

For subsequent analysis, the “Cz” channel was selected as it typically exhibits the strongest CMC in the beta band during TA activity (Ibáñez et al., 2021; Zicher et al., 2023). In the connectivity analysis, the “Cz” signal underwent additional correction to account for the phase shift inherent to the pyramidal tract (Baker et al., 2003). This adjustment was performed by computing and inverting the first derivative of the processed “Cz” signal, a method shown to improve the accuracy of delay estimation between cortical and muscular signals and remove confounding factors (Ibáñez et al., 2021).

#### Spectral analysis

The projection of cortical beta band oscillations to the TA was evaluated using connectivity analysis between the processed “Cz” EEG signal and the CSTs derived as previously described. This analysis was performed using the Neurospec 2.11 toolbox for MATLAB (www.neurospec.org, Halliday, 2015). Coherence was estimated using 1 s signal segments and multitaper spectral estimation (three tapers). The upper 95% confidence limit for significance was determined as $1-0.05^{1/(L-1)}$, where *L* is the number of segments used in the analysis (Rosenberg et al., 1989). To ensure the same coherence significance thresholds across conditions, we cropped all the contraction plateau phases to the same length. This resulted in 49 aligned segments per subject and contraction level, establishing a significance threshold of 0.041.

**Figure 2.**
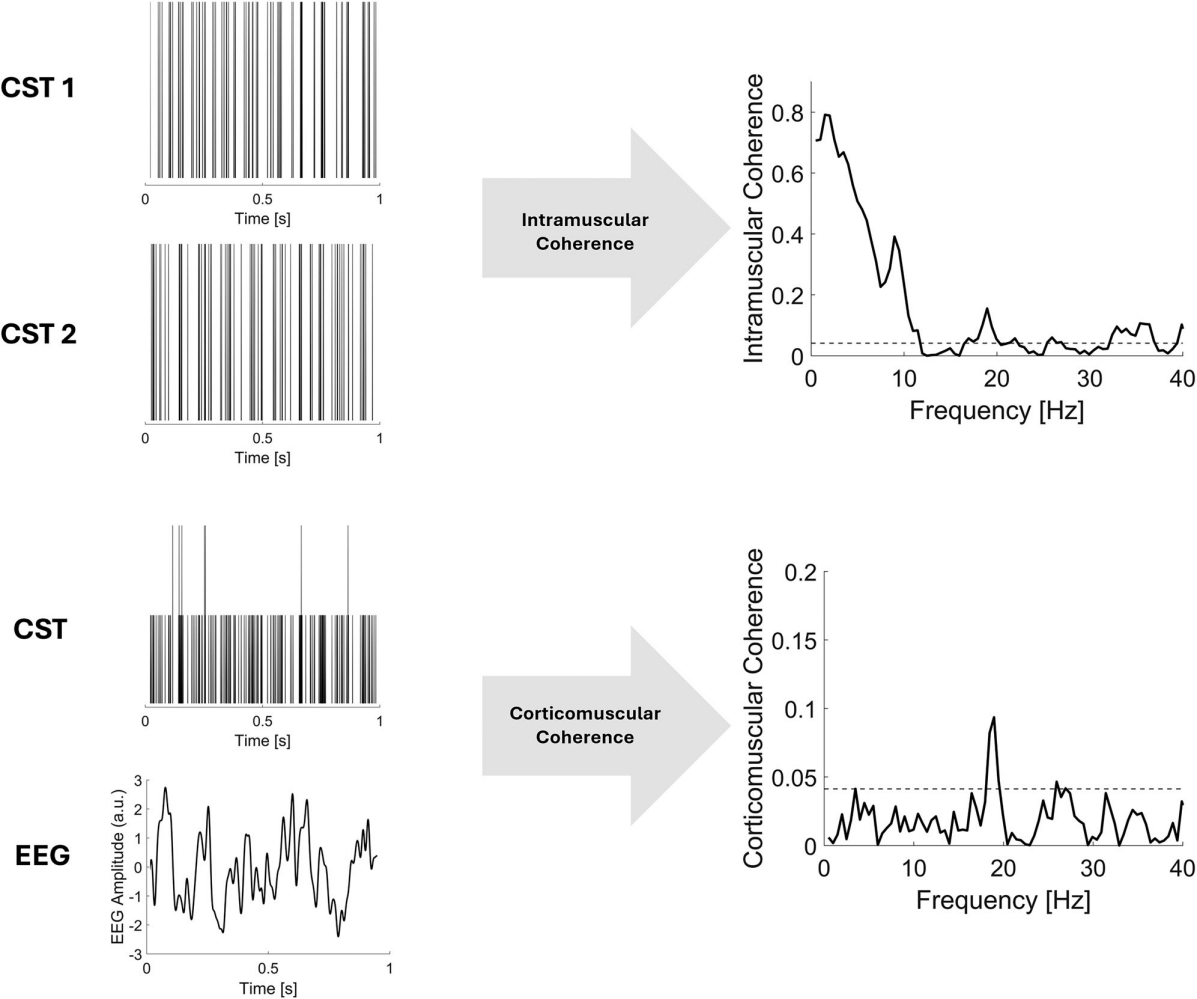
Intramuscular and Corticomuscular Coherence estimation. Representative example of calculation of coherence functions in one subject performing a contraction at 10% MVC. IMC (top) was estimated from the CSTs of two independent subsets of MUs, obtained by evenly splitting the decomposed MU pool. CMC (bottom) was computed between the CST of all MUs identified during the contraction and the EEG signal recorded from the Cz channel.

Intramuscular coherence (IMC) was also computed to investigate its association with CMC. For IMC estimation, the MU pool was divided into two randomly selected subsets of equal size. An example for one subject and the MVC level is shown in Figure 2.

Corticomuscular transmission was further characterized in the time domain using the cumulant density function, which estimates the transmission delay at which significant correlation between cortical and muscular activity peaks (Halliday et al., 1995). Similar to coherence, confidence limits for significant correlation peaks were defined as $\pm \frac{1.96}{\sqrt{R}}$, where *R* represents the number of data points. To investigate corticomuscular transmission delays originating from the brain, the analysis targeted the time point of the highest peak identified at positive lags. If this peak was not statistically significant, no transmission delay was assigned for that subject at the given contraction level.

Power spectral density (PSD) was calculated using Welch's method in MATLAB with the function *pwelch* (4 s window, 75% overlap). This analysis provided insight into the spectral properties of the cortical and muscular signals.

#### Βeta bursting activity

In this study, we define beta oscillations not as a superposition of multiple sinusoids with varying amplitudes within the 13–30 Hz range but rather as a carrier wave within this band whose amplitude is intermittently modulated over time. Beta bursts emerge as brief increases in the amplitude of the carrier wave. This modulation spreads the signal's energy around the carrier frequency, effectively distributing information across the beta band.

**Figure 3.**
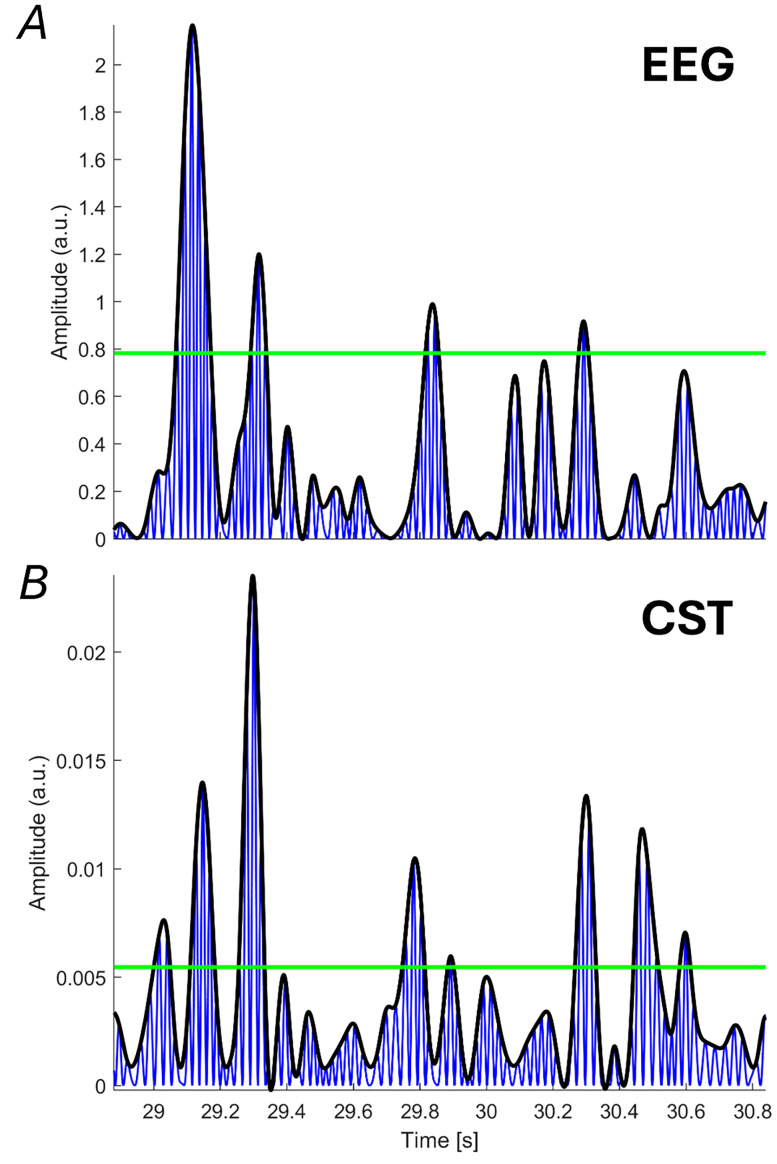
Identification of beta bursts. A thresholding approach is used to detect high-amplitude beta band activity at both the cortical (EEG) and peripheral (CST) levels. The blue trace shows the squared beta band filtered signal (13–30 Hz), the black line represents its envelope, and the green line indicates the threshold used to distinguish burst periods from background activity. ***A***, Example from the squared signal of the EEG channel “Cz”. ***B***, Corresponding example from the CST.

Beta bursting activity was analyzed to assess whether its features varied with force levels or the size of MNs transmitting the common input. Beta bursts in both the EEG signal and the CST were identified using a thresholding method on beta power described by Little et al. (2019). It is important to note that this is only one of several available thresholding methods (Shin et al., 2017; Tinkhauser et al., 2017). Alternative thresholding approaches may produce bursts with different characteristics, e.g., different duration. This analysis considers the fact that beta power is not constant over time and that in some time intervals variations may be relatively fast, so that it is possible to identify short-lived events with high power that are commonly defined as beta bursts. Both signals were bandpass filtered (13–30 Hz) using a fourth-order Butterworth filter. To detect burst events, a thresholding procedure was applied in which the initial threshold—set to the median of the envelope signal—was incrementally increased in steps of 0.25 times the median value, ranging from zero up to six times the median. Their envelopes were segmented into 1 s intervals, and Pearson's correlation coefficient was computed for the power of the envelope and the percentage of the envelope exceeding the threshold. This process was repeated for every given threshold and then averaged across blocks. The optimal threshold was identified as the one with the highest correlation value. Beta bursts were defined as intervals where the signal envelopes exceeded this threshold (Shin et al., 2017; Little et al., 2019). The burst rate was quantified as the average number of bursts per second, while burst duration was calculated as the average time the envelope remained above the threshold. A representative example of beta burst identification is shown in Figure 3.

Following Bräcklein et al. (2022), we classified periods above the threshold as ON events and the intervals between two consecutive ON events as OFF periods. To investigate peripheral burst occurrences in relation to cortical ON events, we identified cortical bursts for each subject and paired them with peripheral windows of equal duration, adjusted for the estimated average transmission delay across subjects at each contraction level calculated using cumulant density analysis. We then averaged peripheral beta activity during these delayed ON windows and compared it to both OFF periods and nondelayed ON windows. To assess the relationship between the identified cortical and peripheral beta activity, we computed Pearson's correlation coefficients for each subject and condition. Importantly, only correlation values with a statistically significant *p* value (*p* < 0.05) were retained for group-level analysis and reporting. Nonsignificant correlations were excluded from further interpretation to focus on robust associations.

#### Statistical analysis

All statistical analyses were conducted using the Jamovi software (www.jamovi.org) and custom-written MATLAB scripts. Results are presented as mean ± SD, with significance set at *p* < 0.05. Data normality was assessed using the Shapiro–Wilk test.

The analyses focused on repeated measures across six contraction levels: 5, 10, 20, 30, 50, and 70% MVC. The effect of the contraction level was investigated for PSD, coherence, transmission delay, burst rate and duration, and related measures using linear mixed models (LMM). For each dependent variable, a LMM was built with the contraction level defined as fixed effect and the subject as random effect. For all LMMs, the fixed-effects omnibus test was used to evaluate the overall influence of contraction levels. When the omnibus test was significant (*p* < 0.05), post hoc pairwise comparisons with Bonferroni’s correction for multiple comparisons were performed to identify specific differences between contraction levels. Post hoc *p* values are provided only for relevant comparisons where not all levels showed significant differences. For tests with *p* < 0.05, the corresponding *F* values are reported. To further evaluate the strength of evidence for or against an effect of contraction level, a Bayesian repeated-measure ANOVA was performed, and the Bayes factor (BF₁₀) was reported. This allowed us to quantify the support for the null model (i.e., no effect of the contraction level) relative to the alternative. The Bayes factors provide a continuous measure of evidence, where values between 1 and 0.33 do not provide evidence in favor of the null hypothesis, values between 0.33 and 0.05 indicate positive evidence for the null hypothesis, and those between 0.05 and 0.006 indicate strong evidence (Kass and Raftery, 1995). This approach thus offers a nuanced quantification of evidence in favor of the absence of an effect, rather than relying solely on nonsignificant *p* values. A linear regression model was used to evaluate the relationship between CMC and IMC. Additionally, a one-way ANOVA was conducted to evaluate significant differences in correlation coefficients between cortical ON bursting events and their corresponding peripheral delayed windows, nondelayed windows, and OFF windows where no bursting event was detected.

## Results

We extracted MU activity for each subject across different force levels, with the number of reliable MUs identified at each level reported in Table 1. To ensure consistency in neural drive estimation, we randomly selected a subset of units at each force level, matching the total number of action potentials analyzed across conditions (see Materials and Methods). This is needed to match the quality of estimate of the neural drive for each contraction level, which otherwise would be an influencing factor in coherence estimates (Negro et al., 2009; Table 1).

**Table 1.** Summary of experimental results

	5% MVC	10% MVC	20% MVC	30% MVC	50% MVC	70% MVC
Decomposed MUs	23.58 ± 6.41	28.41 ± 10.65	28.41 ± 10.55	26.75 ± 10.74	17.33 ± 9.59	7.83 ± 5.02
Included in the analysis MUs	13.75 ± 7.66	12.41 ± 7.22	11.91 ± 6.74	11.51 ± 6.57	9.58 ± 6.70	7.58 ± 5.08
Recruitment thresholds (%)	3.68 ± 0.98	6.72 ± 1.84	13.39 ± 1.43	22.14 ± 3.34	33.82 ± 5.72	48.13 ± 6.07
CMC	0.08 ± 0.02	0.09 ± 0.03	0.08 ± 0.02	0.07 ± 0.03	0.07 ± 0.02	0.08 ± 0.02
CMC transmission frequency (Hz)	21.91 ± 5.23	21.41 ± 5.08	21.50 ± 5.91	21.16 ± 4.82	18.66 ± 4.91	20.41 ± 4.94
IMC	0.23 ± 0.13	0.22 ± 0.11	0.17 ± 0.08	0.18 ± 0.11	0.15 ± 0.05	0.19 ± 0.10
IMC transmission frequency (Hz)	20.83 ± 5.23	29.91 ± 4.33	18.83 ± 4.36	18.83 ± 5.11	20.50 ± 5.35	19.00 ± 3.74
PSD CST (W/Hz)	2.22 ± 1.42	2.44 ± 1.68	2.77 ± 2.27	2.66 ± 1.95	2.28 ± 1.63	2.03 ± 1.13
PSD EEG (W/Hz)	1.83 ± 1.08	1.75 ± 0.74	1.87 ± 1.12	1.74 ± 1.07	1.50 ± 0.51	1.49 ± 0.47
Bursts rate CST (Hz)	3.55 ± 0.76	3.61 ± 0.83	3.72 ± 0.50	3.73 ± 0.92	3.53 ± 0.74	3.32 ± 0.65
Bursts duration CST (ms)	59.46 ± 10.36	58.69 ± 10.74	54.97 ± 5.63	52.26 ± 4.75	64.11 ± 9.67	70.28 ± 10.10
Bursts rate EEG (Hz)	3.18 ± 1.22	3.09 ± 1.27	3.22 ± 1.26	3.12 ± 0.90	3.21 ± 1.19	3.27 ± 0.89
Burst duration EEG (ms)	54.20 ± 8.35	57.52 ± 7.72	56.55 ± 7.37	53.01 ± 4.88	54.80 ± 10.20	54.23 ± 6.71
Transmission delays (ms)	25.99 ± 3.24	26.16 ± 3.36	24.17 ± 3.54	22.77 ± 3.61	21.49 ± 3.16	22.28 ± 2.29

Mean ± SD of experimental results across six contraction levels, expressed as a percentage of MVC. PSD values for EEG are scaled by $10^{-3}$, while those for the CST are scaled by $10^{-4}$.

**Figure 4.**
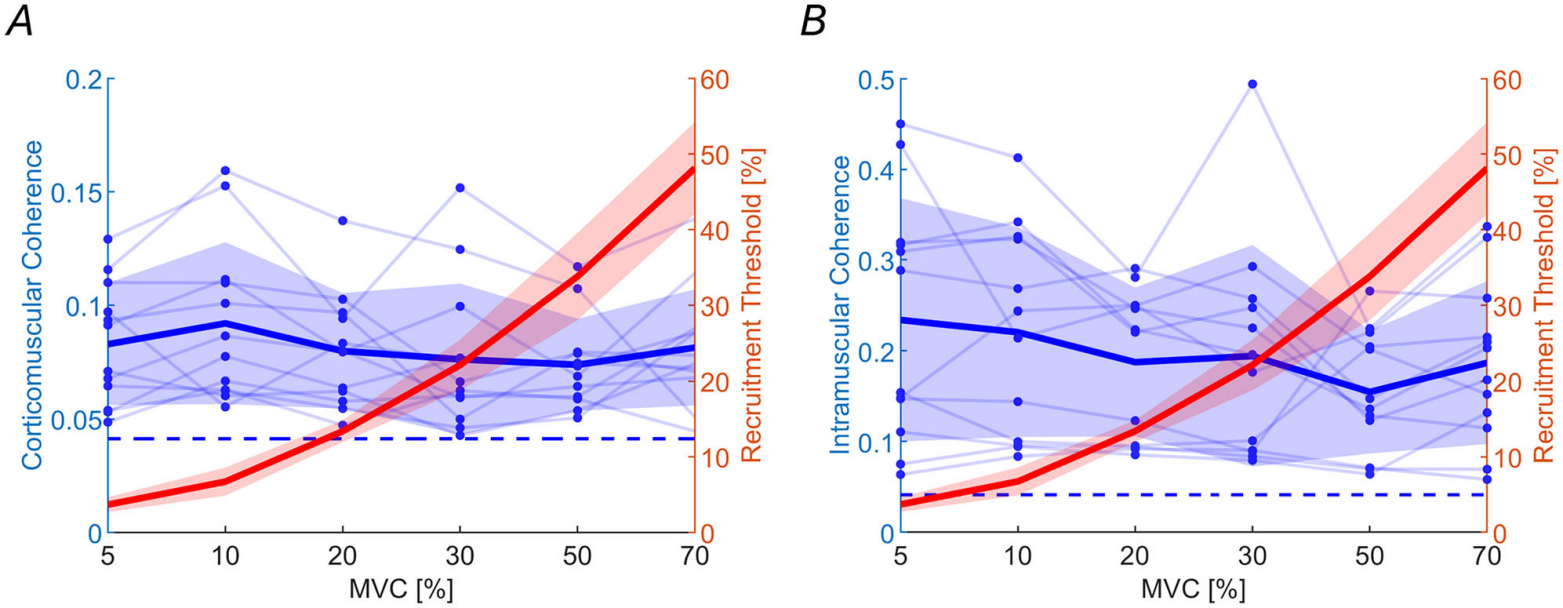
CMC and IMC do not change across different size MUs. ***A***, Maximum CMC and (***B***) maximum IMC values in the beta band range (13–30 Hz) are shown in blue, with individual data points and lines for each subject. Recruitment thresholds of the analyzed MUs for each group are displayed in red. Bold lines indicate average values, while shaded areas represent the SD. The horizontal dashed line marks the statistical significance threshold for the coherence values.

The average recruitment threshold of the MUs decomposed at each MVC level—after applying the quality and standardization procedures described in the Materials and Methods—is shown in red in Figure 4. With increasing levels of MVC, the average recruitment thresholds of the subsets of identified MUs increased (*F*_(5,55)_ = 303; *p* < 0.001; BF₁₀ = 2.9 × 10^40^), as expected (all *p* < 0.001). At each force level, indeed, the decomposition algorithm would tend to identify the MUAPs with highest energy that are usually associated with the MUs with highest thresholds (Farina and Holobar, 2016). According to the size principle (Henneman, 1957), the groups of MUs with different recruitment thresholds corresponded to MNs of different sizes.

To better quantify how the neural dynamics described in the Materials and Methods and analyzed in the Results change across these groups, we report in Table 2 the percentage differences of mean values relative to the 5% MVC baseline. In the following sections, we analyze these neural dynamics in detail.

**Table 2.** Percentage differences in neural dynamics across contraction levels relative to 5% MVC

Percentage difference	5–10% MVC (%)	5–20% MVC (%)	5–30% MVC (%)	5–50% MVC (%)	5–70% MVC (%)
CMC	11.06	−3.63	−8.03	−10.93	−1.82
CMC transmission frequency	−2.28	−1.90	−3.42	−14.82	−6.84
IMC	−4.46	−24.43	−21.37	−33.56	−16.37
IMC transmission frequency	−9.20	−9.60	−9.60	−1.60	−8.80
PSD CST	10.10	25.11	20.17	2.90	−8.18
PSD EEG	−4.33	2.22	−4.48	−17.68	−18.07
Bursts rate CST	1.78	4.99	5.24	−0.44	−6.49
Bursts duration CST	−1.30	−7.55	−12.10	7.82	18.19
Bursts rate EEG	−2.56	1.44	−1.71	1.06	2.94
Burst duration EEG	6.12	4.32	−2.20	1.09	0.04
Transmission delays	0.63	−6.99	−12.41	−17.33	−14.29

To better assess differences across levels, we also report the percentage difference of each feature's mean value at each level relative to the 5% MVC condition (used as the baseline). This provides a clearer quantification of changes across contraction levels, beyond statistical significance alone.

### Cortical beta oscillation are consistently projected to the MN pool

**Figure 5.**
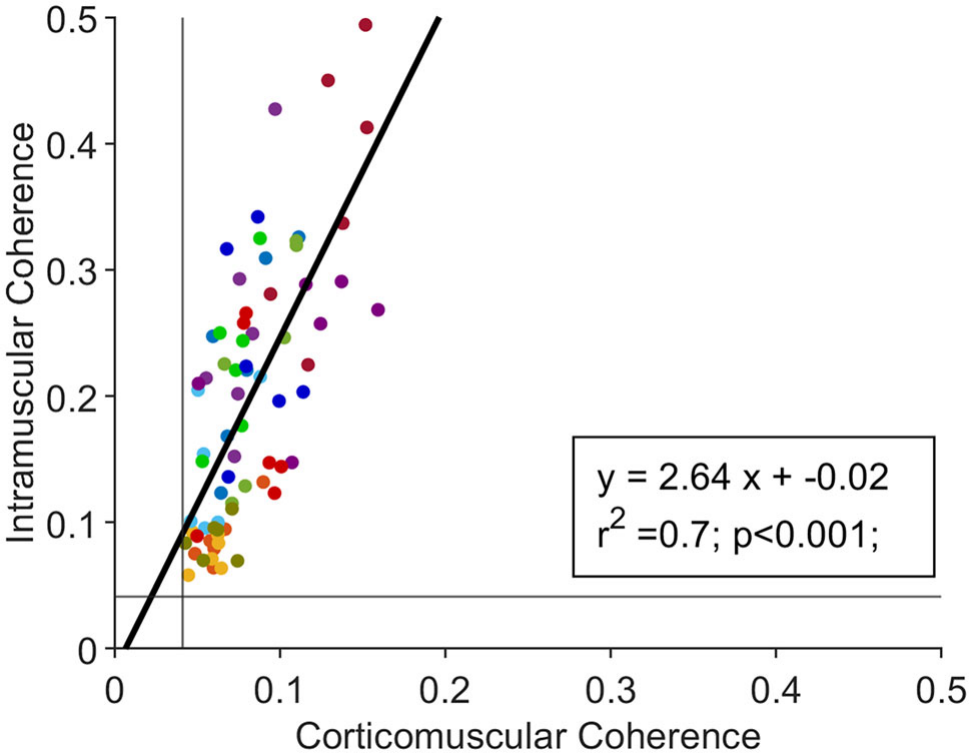
CMC and IMC values are linearly correlated. Each point represents the maximum IMC value in the beta band corresponding to the maximum CMC value for each subject and contraction level. A total of 72 points are shown, color-coded by subject. The continuous light gray lines indicate the statistical significance threshold for coherence, which remains constant across conditions. The linear regression line is also displayed in black.

We analyzed CMC across groups of MNs with different recruitment thresholds. In all subjects, CMC values were statistically significant. CMC values remained constant across groups and conditions (Fig. 4*A*; *F*_(5,55)_ = 1.41; *p* = 0.23; BF₁₀ = 0.32), indicating stable frequency coupling between the cortex and MNs regardless of the MN size. Additionally, no change was observed in the peak transmission frequency of CMC, further supporting the stability of this transmission (*F*_(5,55)_ = 0.63; *p* = 0.67; BF₁₀ = 0.12). Similarly, IMC values were statistically significant across subjects, with no differences observed between MN groups (Fig. 4*B*; *F*_(5,55)_ = 2.08; *p* = 0.08; BF₁₀ = 0.81) nor in their peak transmission frequencies (*F*_(5,55)_ = 0.56; *p* = 0.72; BF₁₀ = 0.10). The similar behavior of CMC and IMC for MN of different sizes was further confirmed by the analysis of the linear correlation between CMC and IMC values in the beta band across subjects and MVC levels (Fig. 5). A strong linear correlation was observed between CMC and IMC (*F*_(12,59)_ = 11.5; *r*^2^ = 0.7; *p* < 0.001), supporting the hypothesis that MN beta power is mainly transmitted through cortical projections.

**Figure 6.**
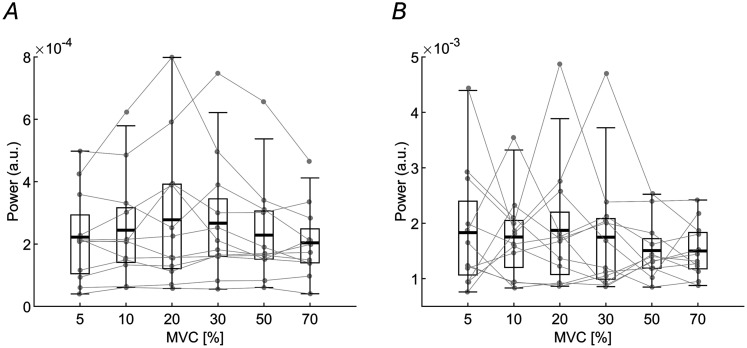
PSD in the beta band across different contraction levels. Each point and line represent one subject. ***A***, Maximum values of the PSD in the beta band (13–30 Hz) across different contraction levels for the analyzed groups of MUs. ***B***, The same analysis is made for the EEG. There are no significant changes.

Because CMC is not directly related to the strength of the beta oscillations, being a normalized measure, we also analyzed the PSD of EEG and the CST in the beta band across the different groups of MUs (Fig. 6). The results showed no significant differences, with beta band power remaining stable in both the CST of the MU groups (Fig. 6*A*; *F*_(5,55)_ = 1.63; *p* = 0.17; BF₁₀ = 0.42) and the EEG recorded from the “*Cz*” channel (Fig. 6*B*; *F*_(5, 55)_ = 0.68; *p* = 0.64; BF₁₀ = 0.12).

**Figure 7.**
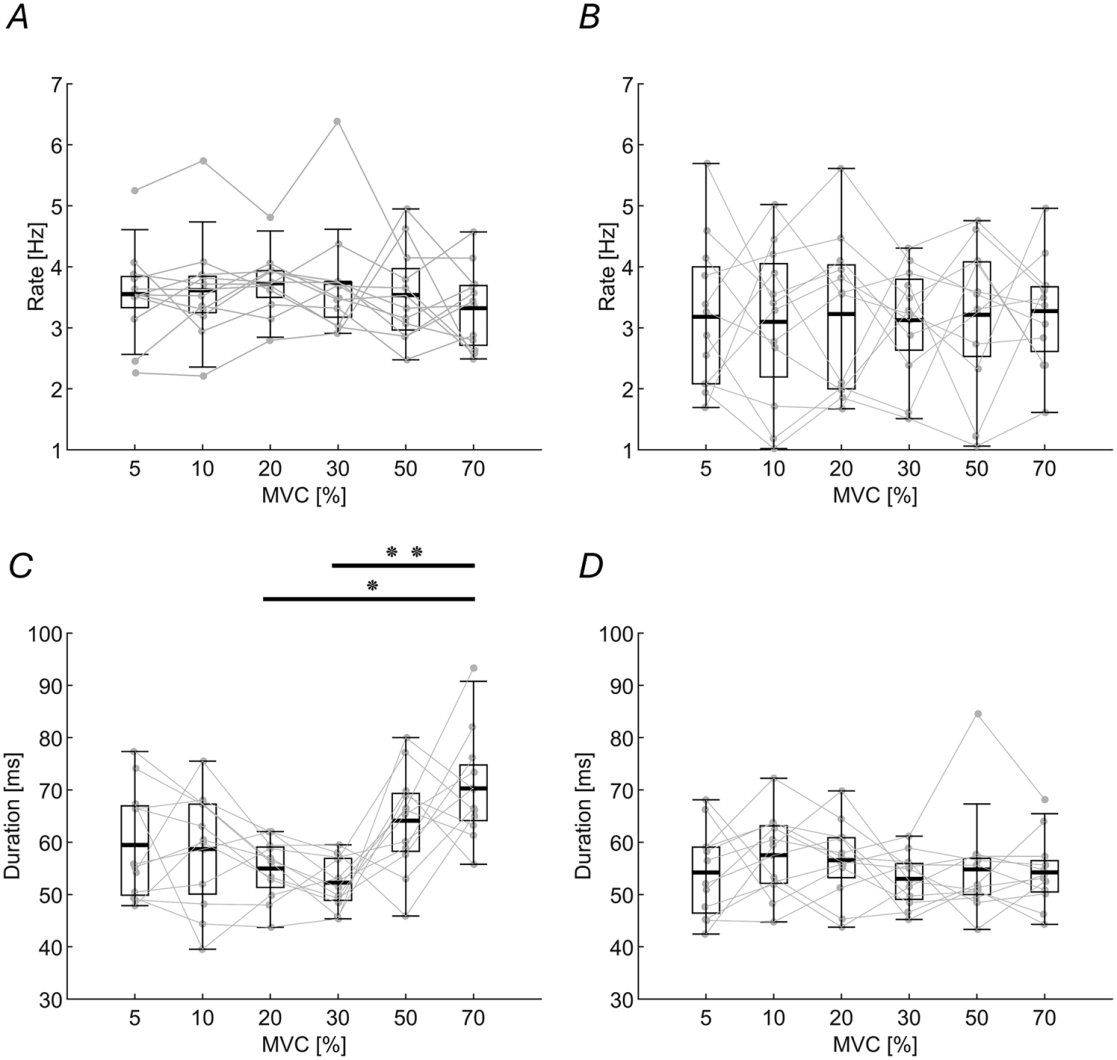
The beta burst rate and duration at cortical and peripheral level across different contraction levels. Each point and line represent an individual subject. This figure illustrates the burst rate and duration values for MU groups and EEG recordings. ***A***, The average burst rate at the MU level. ***B***, The average burst rate at the “Cz” EEG channel. ***C***, Average burst duration at the MU level, showing significant differences between 20 and 70% MVC, as well as between 30 and 70% MVC. ***D***, Average burst duration at the “Cz” EEG channel. *p = 0.001; **p < 0.001.

During sustained contractions, cortical and peripheral activity occurs in bursts (Bräcklein et al., 2022; Echeverria-Altuna et al., 2022). Therefore, in addition to the spectral analysis described above, we also investigated the bursting nature of central and peripheral beta oscillations. To determine whether the features of beta bursts, such as rate and duration, remained consistent across MNs of different sizes, we analyzed their stability. As shown in Figure 7, *A* and *B*, burst rates for both the CST and EEG remained stable across MN groups (*F*_(5,55)_ = 0.97; *p* = 0.44; BF₁₀ = 0.17; and *F*_(5,55)_ = 0.06; *p* = 0.99; BF₁₀ = 0.05, respectively). Burst duration also remained stable for EEG (Fig. 7*D*; *F*_(5,55)_ = 0.64; *p* = 0.67; BF₁₀ = 0.12). However, a significant difference in CST burst duration was observed (Fig. 7*C*; *F*_(5,66)_ = 6.4; *p* < 0.001; BF₁₀ = 883.01), in particular between 20 and 70% MVC (*p* = 0.001) and between 30 and 70% MVC levels (*p* < 0.001). All values for the burst rate and duration are reported in Table 1.

### The fastest corticospinal fibers project beta oscillations to the entire MN pool

**Figure 8.**
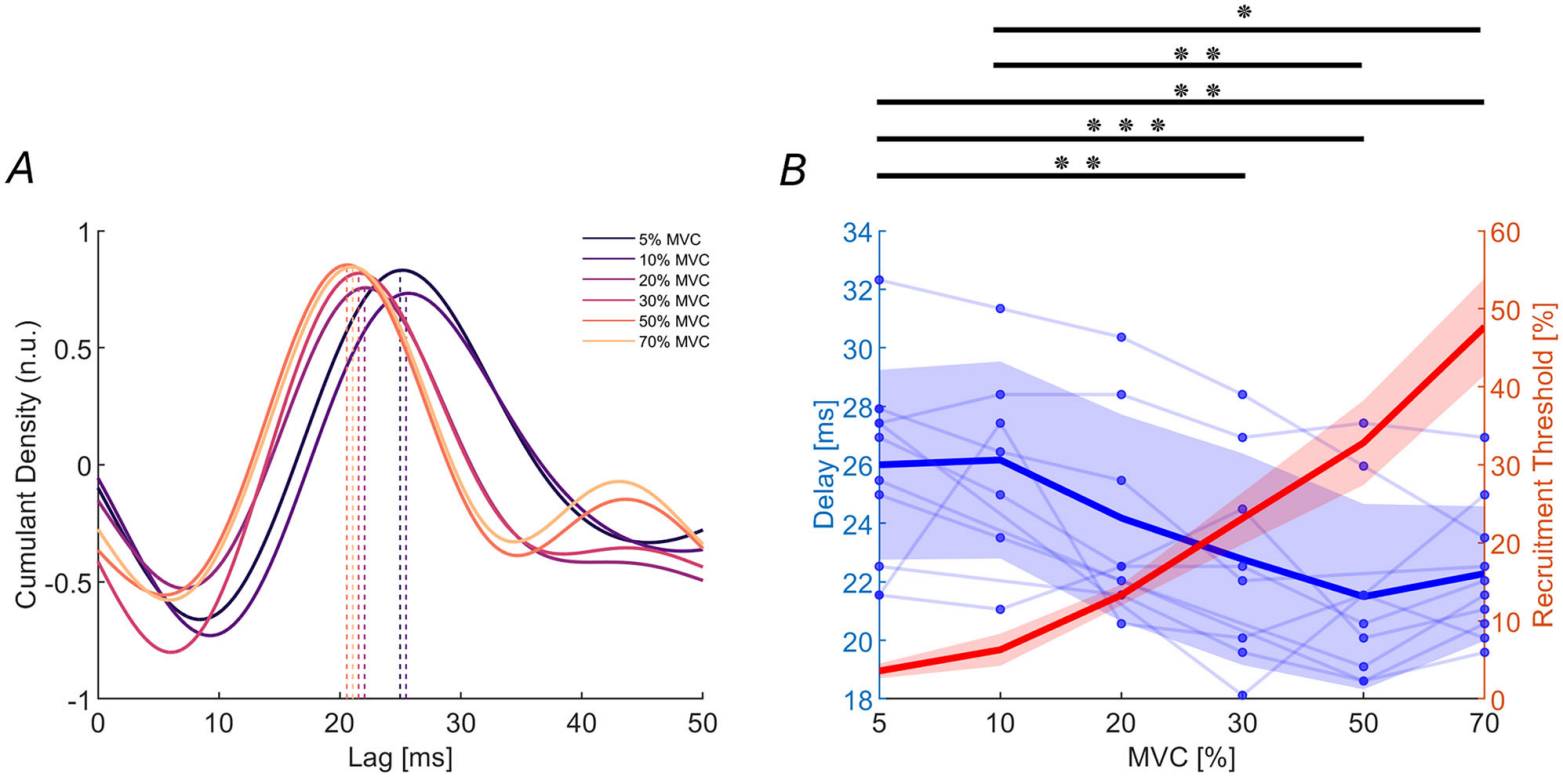
Transmission delays of cortical beta oscillations to the muscle show a significant decrease across MUs groups of different sizes. ***A***, Average normalized cumulant density across subjects for each contraction level between the EEG and the corresponding MU group. The dashed line represents the average peak and transmission delay identified using the cumulant density. ***B***, Transmission delays of beta band oscillations from the cortex to the TA muscle, estimated using the cumulant density function, are shown in blue across different contraction levels, with individual subject data points and lines. The recruitment thresholds for the pooled MUs at each contraction level are shown in red. Solid lines indicate average values, while shaded areas represent the SD. *p = 0.025; **p < 0.01; ***p < 0.001.

We further examined the transmission delay between the cortex and muscle. While previous findings showed that the fastest corticospinal fibers contribute to beta band CMC at low contraction levels (10% MVC; Ibáñez et al., 2021), there are no previous results on beta transmission delays for higher-threshold MNs. Here, we estimated the transmission delays with cumulant density analysis across contraction levels. The average cumulant density from the TA revealed a distinct positive peak in most individual traces at a positive time lag (Fig. 8*A*). Significant cumulant density peaks were detected in all subjects and in 54 out of 72 analyzed blocks.

The results revealed a significant reduction in transmission delay as the contraction intensity increased (Fig. 8*B*; *F*_(5,38.1)_ = 8.14; *p* < 0.001). The mean delays ranged from 26.0 ± 3.2 ms at 5% MVC to 22.3 ± 2.3 ms at 70% MVC, with intermediate values of 26.2 ± 3.4 ms (10% MVC), 24.2 ± 3.5 ms (20% MVC), 22.8 ± 3.6 ms (30% MVC), and 21.5 ± 3.2 ms (50% MVC). Significant differences in delay were observed between 5 and 30% MVC (*p* = 0.007); 5 and 50% MVC (*p* < 0.001); 5 and 70% MVC (*p* = 0.002); 10 and 50% MVC (*p* = 0.004); 10 and 70% MVC (*p* = 0.025; Fig. 8*B*). However, these differences in delay were fully compatible with the distribution of spinal MN axonal conduction velocities. Using an approximate average length of 500 mm for the axons of lower MNs innervating the TA and axonal conduction velocities ranging from 41 m/s at 5% MVC to 57.5 m/s at 70% MVC (Kakuda et al., 1992), the spinal MN axonal transmission delay varied between ∼12.2 ms for lower-threshold MNs and 8.7 ms for higher-threshold ones. This range of delays explains the range observed in estimates of corticomuscular delays, which resulted as the sum of a constant corticospinal delay of ∼14 ms and a delay that depended on the variability in peripheral axonal conduction velocities (with the values reported above for low- and high-threshold MNs). This result supported the hypothesis that beta inputs drive all MNs similarly, regardless of their size, through the fastest fibers of the corticospinal tract.

**Figure 9.**
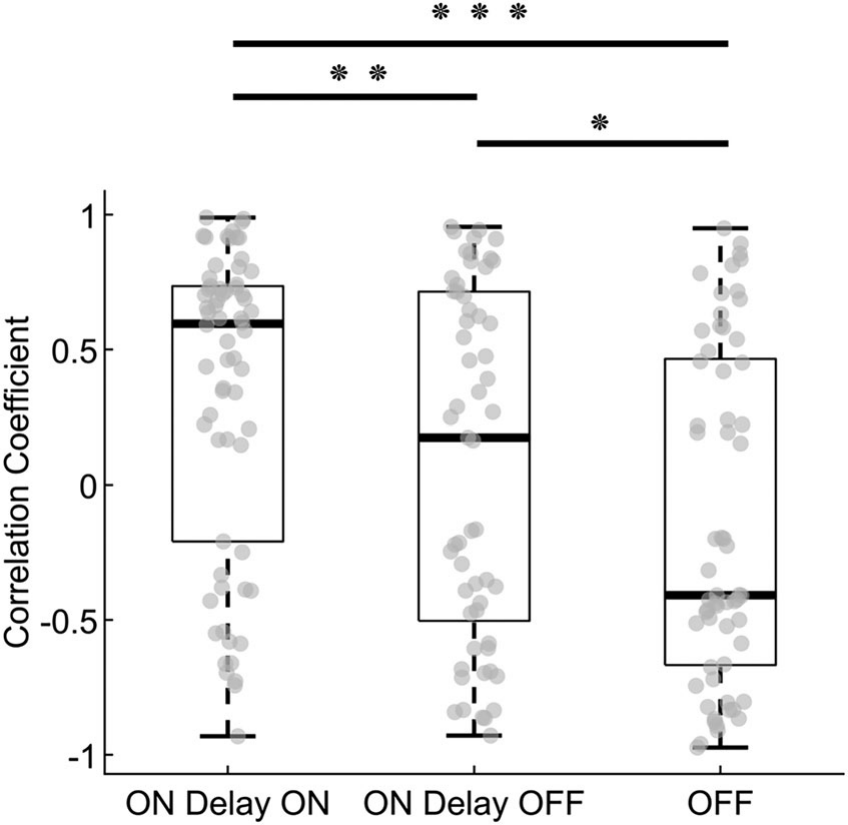
Correlation of cortical beta burst windows with peripheral delayed, nondelayed, and OFF windows. Significant correlation coefficients between cortical and corresponding peripheral burst windows for each contraction level and subject are shown in gray. Significant differences were found between cortical ON windows delayed by the average transmission delay across subjects for that contraction level (ON Delay ON) and nondelayed ON windows (ON Delay OFF), as well as between ON Delay ON windows and peripheral windows where no cortical burst occurred (OFF). *p = 0.043; **p = 0.039; ***p < 0.001.

Finally, we extended our analysis to predict peripheral beta bursts appearance based on cortical bursts, accounting for the estimated average transmission delay between the cortex and periphery. The reported burst rates for CST and EEG were comparable in number, supporting the idea that cortical bursts are transmitted to tonically contracted muscles, as previously reported (Bräcklein et al., 2022). For each subject, we identified cortical bursts and paired them with peripheral windows of equal duration, adjusted for the estimated average transmission delay at each contraction level (see Materials and Methods). We then calculated the correlation between these cortical bursts and their corresponding delayed ON windows, comparing them to OFF periods and nondelayed ON windows. Only significant correlation values were reported across subjects. Notably, 88.9% of blocks showed significant correlations for delayed ON windows, 79.1% for nondelayed ON windows, and 84.7% for OFF windows. Since we accounted for transmission delays at each MVC level and observed no significant differences in beta correlation or power between the cortex and TA across MVC levels (as shown in the results above), all averaged correlation coefficients were pooled across the three conditions. The median and SD of correlation coefficients were 0.59 ± 0.56 for delayed ON windows, 0.17 ± 0.64 for nondelayed ON windows, and −0.40 ± 0.60 for OFF periods. The analysis revealed that correlation values for delayed ON windows were significantly greater than those for nondelayed ON windows (*χ*^2^(1) = 4.24; *p* = 0.039) and OFF periods (*χ*^2^(1) = 18.2; *p* < 0.001). A significant difference was also found between correlation coefficients of nondelayed ON windows and OFF periods (*χ*^2^(1) = 4.09; *p* = 0.043; Fig. 9). These findings further support that cortical beta bursts are transmitted to the periphery with delays that align with estimates from the cumulant density analysis.

## Discussion

This study is the first to examine the corticospinal transmission of beta oscillations across distinct MN groups with markedly different recruitment thresholds, spanning nearly the entire force range of the muscle. Our findings demonstrate that cortical beta oscillations are transmitted similarly across the MN pool, regardless of the MN size. The estimated transmission delays align with the fastest corticospinal pathways. This transmission remains consistent across different contraction levels, as confirmed by PSD analysis.

These results suggest that corticospinal fibers conveying beta oscillations engage all MNs similarly, utilizing the minimal delay enabled by the fastest pathways. Since CMC does not decrease with increasing recruitment threshold, it is likely that the corticospinal tract maintains its projection not only to low-threshold MUs but also to high-threshold ones. This finding challenges the assumption that high-threshold MNs—primarily recruited during strong contractions and largely controlled by the reticulospinal tract—receive less corticospinal input. Instead, our results indicate that beta band oscillations, like low-frequency common input involved in force control, are consistently transmitted to the entire MN pool, reinforcing the widespread role of the corticospinal tract in motor control. However, we emphasize that these findings pertain specifically to the transmission of beta band cortical oscillations. They do not preclude additional or complementary influences from other descending pathways—such as the reticulospinal tract or spinal afferent fibers—particularly during high-force tasks, where their contribution may be functionally distinct from the corticospinal input analyzed here.

### Projection of cortical beta oscillations across MNs of different sizes

We identified a linear relationship between CMC and IMC values, suggesting that the primary source of peripheral beta oscillations is cortical activity. This finding aligns with previous studies demonstrating synchronization between cortical and peripheral beta oscillations (Ibáñez et al., 2021; Bräcklein et al., 2022). When the curve is populated with sufficient data from individual subjects, distinct clusters emerge, with each cluster corresponding to an individual. This observation supports earlier findings that CMC values vary between subjects (Matsuya et al., 2017), despite being unaffected by different MVC levels (see Results). Beyond confirming the cortical origin of peripheral beta oscillations, the linear CMC–IMC association implies that, with sufficient data, it may be possible to estimate CMC from IMC.

To accurately assess the corticomuscular transmission of beta oscillations across the MN pool, we accounted for and mitigated potential confounding factors in both cortical EEG signals and peripheral surface EMG signals—including phase correction to the former and MU decomposition to the latter. This approach enabled us to investigate whether beta band input to the MN pool is transmitted similarly to spinal MNs, irrespective of their size, comparable to the behavior observed for low-frequency inputs associated with force control. Our findings revealed that CMC remained constant and independent of the MN size. Furthermore, the PSD and bursting activity of beta oscillations were predominantly consistent across the MN pool.

### The fastest corticospinal fibers project to the entire MN pool

Based on the measured neural dynamics, we concluded that cortical beta oscillations are similarly projected across the MN pool. If this corticomuscular transmission is truly independent of the MN size, it prompts further questions about the organization of upper MNs and their connectivity to the entire pool. To address this, we calculated the transmission delays of cortical beta oscillations across pools of MNs of varying sizes and observed a decrease in delay as the MN size increased.

At low contraction levels, the transmission delays from the cortex to muscle aligned closely with values previously reported using motor-evoked potentials (Rossini et al., 1999; Cantone et al., 2019). These delays are compatible only with the fastest corticospinal fibers transmitting cortical beta oscillations, consistent with prior findings (Ibáñez et al., 2021). However, these conclusions could previously be drawn only for low-threshold MUs.

Accounting for the peripheral axonal delays, which determine faster spinal–muscle transmission for larger MNs (see Results), our findings suggest that the corticospinal tract transmission delay remained constant. This consistent corticospinal transmission delay indicates that the same fastest corticospinal fibers projecting cortical beta oscillations to smaller MNs are also responsible for transmitting these oscillations to larger MNs.

In summary, the central pathway delay is remarkably short, aligning with the conduction speed of the fastest corticospinal fibers, for both small and large MNs.

### Transmission of cortical beta bursts to the periphery

At the cortical level, the beta burst rate and duration remained consistent across different contraction levels. However, while the burst rate was constant across the MN pool, burst duration showed a significant increase between both 20 and 70% MVC, as well as 30 and 70% MVC. This is the only characteristic of beta oscillations and bursts that varied with the MN size. While we cannot exclude physiological reasons, this variation can also be due to methodological limitations in estimating bursts durations in the two signals, with a situation of relatively poor signal-to-noise ratio.

We then analyzed transmission of cortical bursts to the muscle. While Bräcklein et al. (2022) previously examined activity around cortical beta bursts, they did not include delay estimates. Here, we incorporated transmission delays, estimated using cumulant density, which varied with contraction levels. By applying a burst detection threshold to identify ON periods in cortical beta activity and estimating peripheral burst onsets using average transmission delays derived from the cumulant density analysis, we observed a stronger correlation between cortical and peripheral bursts compared with nondelayed ON periods and to OFF periods. This suggests that the transmission delays calculated here are likely accurate, as they identified time shifts that significantly increased the correlation between bursts at cortical and spinal levels. More importantly, this finding implies that the fastest corticospinal fibers projecting to the entire MN pool are also likely the primary pathway for cortical burst transmission. It is important to note, however, that the analysis of ON and OFF periods does not imply that all ON periods are transmitted to the periphery, while OFF periods (defined as intervals with lower beta power) are not. Rather, the analysis is based on averaging peripheral beta activity time-locked to delay-corrected cortical ON periods and doing the same for OFF periods. The observation that peripheral beta activity is greater during cortical ON periods than during OFF periods offers an alternative way to quantify the correlation between fluctuations in cortical and peripheral beta power. Future analyses could benefit from advanced burst detection algorithms capable of distinguishing bursts based on their waveform rather than a single amplitude threshold. Recent research has linked burst waveform characteristics at the cortical level to specific functions in movement-related cortical dynamics (Szul et al., 2023), suggesting that analyzing the specific waveforms of cortical bursts could help determine whether only a subset of them are transmitted to the periphery. Such approach could refine our understanding of the transmission dynamics between cortical and peripheral beta bursts.

### Limitations

The identification of heterogeneous and particularly large MUs remains a challenge due to the limitations of the EMG decomposition algorithm, particularly due to the increased cancellation of interference EMG signals at higher contraction levels. While larger MUs were successfully decomposed at submaximal contraction levels, even larger MUs likely remain to be identified at higher force levels—though such efforts are challenging both technically and due to muscle fatigue. Moreover, although the transmission delays at lower MVCs are consistent with values reported in the literature, it is important to note that the cumulant density function provides only a rough estimate of these delays, as it is sensitive to noise. Finally, all results were obtained for the TA muscle, and while beta band oscillations are ubiquitous, generalizing these findings to other muscles is not possible at present. Different muscles exhibit not only distinct anatomical features but also unique neural control strategies (Ushiyama et al., 2010). To determine the broader applicability of these conclusions, further studies using similar methods should be conducted across a variety of muscles.
